# Supplementary material for: Enhanced Astrocyte Activity and Excitatory Synaptic Function in the Hippocampus of Pentylenetetrazole Kindling Model of Epilepsy
Source: Int J Mol Sci. 2023 Sep 25;24(19):14506. doi: 10.3390/ijms241914506 (PMC10572460; doi:10.3390/ijms241914506)
Supplement: Supplementary file 1 [file ijms-24-14506-s001.zip › ijms-2608056-supplementary.pdf]

Supplementary Table S1. Comparative results between experimental groups for each experiment presented.

PARAMETER	EXPERIMENTAL GROUP	VALUE (MEAN ± S.E.M.)	N	P-VALUE	FIGURE
**Incidence of ADs (min^{-1})**	PTZ D1	0.00883 ± 0.00532	5 mice	0.0088 (D1 vs. D13)	1D
	PTZ D13	0.03800 ± 0.00565			
**Latency of ADs (s)**	PTZ D1	426.3 ± 107.5	5 mice	0.0480 (D1 vs. D13)	1D
	PTZ D13	109.8 ± 37.7			
**Duration of ADs (s)**	PTZ D1	21.0 ± 0.4	5 mice	0.0466 (D1 vs. D13)	1D
	PTZ D13	29.4 ± 2.2			
**GFAP IF**	Control	2.41 ± 0.22	7 mice	< 0.0001 (Control vs. PTZ)<br>0.9580 (Control vs. VEH)<br>< 0.0001 (PTZ vs. VEH)	2A
	VEH	2.85 ± 0.40	8 mice		
	PTZ	24.54 ± 1.42	8 mice		
**GFAP WB**	Control	0.49 ± 0.04	6 mice	0.0077 (Control vs. PTZ)	2B
	PTZ	0.61 ± 0.01	6 mice		
**Transients Duration (s)**	Control	20.1 ± 0.49	179 astrocytes	< 0.0001 (Control vs. PTZ)<br>> 0.9999 (Control vs. VEH)<br>< 0.0001 (PTZ vs. VEH)	3D
	VEH	20.3 ± 0.44	185 astrocytes		
	PTZ	23.9 ± 0.60	155 astrocytes		
**Slow Transients Oscillations (%)**	Control	30.0 ± 1.5	163 astrocytes	0.0090 (Control vs. PTZ)<br>> 0.9999 (Control vs. VEH)<br>0.0055 (PTZ vs. VEH)	3D
	VEH	30.0 ± 1.3	180 astrocytes		
	PTZ	37.2 ± 1.7	154 astrocytes		
**FT/ST index**	Control	4.81 ± 0.47	163 astrocytes	0.0090 (Control vs. PTZ)<br>> 0.9999 (Control vs. VEH)<br>0.0055 (PTZ vs. VEH)	3D
	VEH	4.82 ± 0.44	180 astrocytes		
	PTZ	3.28 ± 0.34	154 astrocytes		
**sEPSCs Frequency (Hz)**	Control	0.70 ± 0.14	10 neurons	0.0327 (Control vs. PTZ)<br>0.9962 (Control vs. VEH)<br>0.0401 (PTZ vs. VEH)	4A
	VEH	0.73 ± 0.09	10 neurons		
	PTZ	1.47 ± 0.26	14 neurons		
**sEPSCs Amplitude (pA)**	Control	13.90 ± 0.95	10 neurons	0.9695 (Control vs. PTZ)<br>0.2971 (Control vs. VEH)<br>0.1621 (PTZ vs. VEH)	4A
	VEH	16.21 ± 1.22	10 neurons		
	PTZ	13.56 ± 0.90	14 neurons		
**sIPSCs Frequency (Hz)**	Control	3.82 ± 0.35	18 neurons	0.9839 (Control vs. PTZ)<br>0.9542 (Control vs. VEH)<br>0.9907 (PTZ vs. VEH)	4B
	VEH	3.68 ± 0.22	12 neurons		
	PTZ	3.74 ± 0.34	16 neurons		
**sIPSCs Amplitude (pA)**	Control	27.63 ± 2.73	18 neurons	0.9954 (Control vs. PTZ)<br>0.5691 (Control vs. VEH)<br>0.5333 (PTZ vs. VEH)	4B
	VEH	31.07 ± 2.66	12 neurons		
	PTZ	27.35 ± 1.15	16 neurons		
**Slope fEPSP (µV/ms)**	Control	1.54 ± 0.06	9 slices	0.0340 Control vs. PTZ)<br>> 0.0500 (Control vs. VEH)<br>< 0.0500 (PTZ vs. VEH)	5A
	VEH	1.38 ± 0.08	5 slices		
	PTZ	3.91 ± 0.40	5 slices		
**Ps Threshold (µV)**	Control	0.35 ± 0.04	9 slices	0.0380 (Control vs. PTZ)<br>0.2928 (Control vs. VEH)<br>0.0470 (PTZ vs. VEH)	5A
	VEH	0.27 ± 0.02	6 slices		
	PTZ	0.17 ± 0.06	5 slices		
**eEPSCs PPR**	Control	1.46 ± 0.13	10 neurons	0.0011 (Control vs. PTZ)<br>0.7026 (Control vs. VEH)<br>0.0053 (PTZ vs. VEH)	5B
	VEH	1.36 ± 0.06	13 neurons		
	PTZ	0.97 ± 0.07	14 neurons		
**eIPSCs PPR**	Control	0.73 ± 0.08	10 neurons	0.5396 (Control vs. PTZ)<br>0.9444 (Control vs. VEH)<br>0.7051 (PTZ vs. VEH)	5B
	VEH	0.70 ± 0.05	13 neurons		
	PTZ	0.64 ± 0.04	14 neurons		
**E/I Amplitude**	Control	0.82 ± 0.10	9 neurons	0.0006 (Control vs. PTZ)<br>0.6179 (Control vs. VEH)<br>0.0047 (PTZ vs. VEH)	5C
	VEH	1.12 ± 0.11	10 neurons		
	PTZ	2.21 ± 0.37	9 neurons		
**E/I Area**	Control	0.11 ± 0.03	9 neurons	0.0247 (Control vs. PTZ)<br>0.9326 (Control vs. VEH)<br>0.0460 (PTZ vs. VEH)	5C
	VEH	0.13 ± 0.03	10 neurons		
	PTZ	0.32 ± 0.03	9 neurons		
**fEPSP PPR**	VEH	1.51 ± 0.09	12 slices	0.0004 (VEH vs. VEH+FC)<br>0.0155 (VEH vs. PTZ)<br>0.2594 (VEH vs. PTZ+FC)<br>< 0.0001 (PTZ vs. PTZ+FC)	6
	VEH + FC	1.80 ± 0.10	12 slices		
	PTZ	1.01 ± 0.05	9 slices		
	PTZ + FC	1.30 ± 0.05	9 slices		

***ADs:** After Discharges; **FT:** Fast Transients; **ST:** Slow Transients; **IF:** Immunofluorescence; **WB:** Western Blot; **EPSCs:** Excitatory Postsynaptic Currents; **IPSCs:** Inhibitory Postsynaptic Currents; **fEPSP:** Field Excitatory Postsynaptic Potentials; **Ps:** Population-Spike; **PPR:** Paired-Pulse Ratio; **PTZ:** Pentylenetetrazole; **VEH:** Vehicle; **FC:** Fluorocitrate; **D:** Day.*
